# Translating theory into practice: assessing the privacy implications of concept-based explanations for biomedical AI

**DOI:** 10.3389/fbinf.2023.1194993

**Published:** 2023-07-05

**Authors:** Adriano Lucieri, Andreas Dengel, Sheraz Ahmed

**Affiliations:** ^1^ Smart Data and Knowledge Services (SDS), Deutsches Forschungszentrum für Künstliche Intelligenz (DFKI) GmbH, Kaiserslautern, Germany; ^2^ Computer Science Department, RPTU Kaiserslautern-Landau, Kaiserslautern, Germany

**Keywords:** deep learning, biomedical image analysis, explainable AI, concept-based explainability, attribution maps, adversarial machine learning, membership inference attack, differential privacy

## Abstract

Artificial Intelligence (AI) has achieved remarkable success in image generation, image analysis, and language modeling, making data-driven techniques increasingly relevant in practical real-world applications, promising enhanced creativity and efficiency for human users. However, the deployment of AI in high-stakes domains such as infrastructure and healthcare still raises concerns regarding algorithm accountability and safety. The emerging field of explainable AI (XAI) has made significant strides in developing interfaces that enable humans to comprehend the decisions made by data-driven models. Among these approaches, concept-based explainability stands out due to its ability to align explanations with high-level concepts familiar to users. Nonetheless, early research in adversarial machine learning has unveiled that exposing model explanations can render victim models more susceptible to attacks. This is the first study to investigate and compare the impact of concept-based explanations on the privacy of Deep Learning based AI models in the context of biomedical image analysis. An extensive privacy benchmark is conducted on three different state-of-the-art model architectures (ResNet50, NFNet, ConvNeXt) trained on two biomedical (ISIC and EyePACS) and one synthetic dataset (SCDB). The success of membership inference attacks while exposing varying degrees of attribution-based and concept-based explanations is systematically compared. The findings indicate that, in theory, concept-based explanations can potentially increase the vulnerability of a private AI system by up to 16% compared to attributions in the baseline setting. However, it is demonstrated that, in more realistic attack scenarios, the threat posed by explanations is negligible in practice. Furthermore, actionable recommendations are provided to ensure the safe deployment of concept-based XAI systems. In addition, the impact of differential privacy (DP) on the quality of concept-based explanations is explored, revealing that while negatively influencing the explanation ability, DP can have an adverse effect on the models’ privacy.

## 1 Introduction

The upcoming implementation of the European Artificial Intelligence (AI) Act (Commission, 2021) will have a considerable impact on the requirements posed on the transparency and interpretability of AI-based systems used in a wide range of biomedical technology and healthcare domains. Recent progress in the field of explainable AI (XAI) provides AI developers and users with a variety of methods and modalities that can help to interpret decision-making processes and validate system functions (Tjoa and Guan, 2020). However, there is still no standardized procedure for the explanation of Deep Learning (DL) models. Regulations such as the General Data Protection Regulation (GDPR) (Council of the European Union, 2016) additionally require that the privacy of all involved data subjects is ensured throughout all deployment phases. Moreover, model privacy is crucial to secure the intellectual property of service providers when hosting AI-based solutions. With more and more promising AI systems being developed by industry and research, it is becoming ever more important to assure that their deployment bears no unforeseen risks for the public and all involved stakeholders. The occurrence of such potential risks can have particularly serious consequences in high stakes application domains such as healthcare or autonomous driving.

The problems of transparency and privacy are traditionally regarded as separate approaches in the research field of AI. However, the goals of both directions are strictly opposing in the context of data-driven algorithms. While transparency and explainability aim at revealing more information about a particular decision or the overall decision-making process, privacy-preserving machine learning tries to focus on dataset-wide statistics, without allowing too much insight on particular decision paths. Several recent studies revealed that both explainability and privacy affect each other significantly in practical deployment. While many works (Milli et al., 2019; Aïvodji et al., 2020; Shokri et al., 2021; Duddu and Boutet, 2022) found that revealing explanations can pose a severe security risk, others (Bozorgpanah et al., 2022; Saifullah et al., 2022) found that adding privacy can significantly diminish the quality of attribution maps.

The most commonly applied explanation methods are based on input feature attribution. These methods operate on the feature level, generating explanations in the input space. This introduces strong limitations due to the often temporal or spatial nature of the input samples. In complex problem settings, as often found in biomedicine, this leads to the inability to explicitly draw attention to feature interactions or higher-level relationships relevant to the decision-making. This is particularly relevant in the case of overlapping and distributed biomarkers (such as colors, shapes, and textures within a tissue). More advanced explanation approaches operate in a more abstract human-centered concept space. These concept-level explanations can provide more diverse and nuanced explanations to users (Lucieri et al., 2022) and have already been shown to have positive effects on the practical utility of AI in the clinical context (Chanda et al., 2023). By closing the interpretation gap between issued explanations and the AI system’s users, human-centered explanations play a major role in increasing the utility of XAI in practice and fulfilling the requirements posed by the AI Act and other regulatory requirements. However, their increasing relevance raises important questions about the impact they have on the privacy of an AI system. Most previous works investigating the interdependency of privacy and explainability focused on low-level XAI methods based on input feature attribution (Milli et al., 2019; Shokri et al., 2021; Saifullah et al., 2022), while Montenegro et al. (2022) investigated privacy-preserving case-based explanations. We argue that more complex XAI methods have received too little attention when it comes to an assessment of their implication on privacy.

This work attempts to fill this gap by assessing if and to which degree concept-based explanations amplify or mitigate the risk of privacy leakage. The aim is to quantitatively measure changes in the theoretical and practical vulnerability of AI models, when issuing explanations of varying complexity while being exposed to Membership Inference Attacks (MIAs). Moreover, the usefulness of privacy-preserving machine learning (i.e., Differential Privacy (DP)) as a defense mechanism and its impact on the quality of issued explanations are investigated. For this purpose, MIAs were applied to three different model architectures, each trained on skin lesion analysis, diabetic retinopathy detection, as well as a synthetic object recognition task. MIAs of different complexities are applied to measure the vulnerability of models in suboptimal and optimal deployment scenarios. Differentially private model variants are trained for each task to assess the impact on attack vulnerability and explanation quality. To the best of our knowledge, this is the first work to investigate the relationship between concept-based explanations, DP, and privacy leakage.

The contributions of this work are as follows.

• An upper bound of the theoretical impact of concept-based explanations on the success of metric- and classifier-based membership inference attacks is empirically determined, finding that concept-based explanations indeed lead to the potential increase in privacy vulnerability.
• Experiments on the practical impact of concept-based explanations revealed that the actual increase in risk is negligible for realistic deployment scenarios.
• Differential privacy fails to defend against membership inference attacks in suboptimal deployment scenarios, and can even reinforce vulnerabilities.
• There is a strong need for differentially private concept-based explanation methods.
• Differential privacy is found to negatively influence the computation of CAVs for explanation.

The remainder of this paper is structured as follows: Section 2 provides the relevant background, as well as an outline of the used datasets and the experimental setting. First, membership inference attacks and differential privacy are introduced, followed by the most relevant XAI methods and an overview of the related work in the intersection of privacy and explainability. The three datasets used in experimentation are then described, followed by a detailed outline of used classification models, training procedures for classifiers and concept-based explanation vectors, as well as the experimental setups for attribution computation and membership inference attacks. The result section (Section 3) reports all the performances of the different classification models, followed by the results of membership inference attacks in theoretical and practical scenarios, when providing different degrees of explanation outputs. In addition, the effect of differential privacy on the computation of concept activation vectors is quantified. All results and corresponding findings are discussed in Section 4, followed by a discussion of the limitations. The paper is concluded in Section 5, summarizing the findings and providing an outlook to potential future work.

## 2 Methodology

The following section outlines all relevant background to the conducted analysis. First, relevant topics including membership inference attacks (MIA), differential privacy (DP), and eXplainable AI (XAI) are introduced. Then, a brief overview of related works investigating the impact of privacy and explainability methods is given. Afterward, the datasets utilized in this study are described and detailed information on the experimental setups is provided.

### 2.1 Membership inference attacks

Membership Inference Attacks have been introduced by Shokri et al. (2017) and refer to a class of attacks that aim to determine if a particular data point was used for the training of a machine learning model. These attacks exploit the privacy vulnerability that can arise when machine learning models are trained on sensitive data. Let *f*_*victim*_(*x*) be the victim model, trained on a private dataset $D_{\mathrm{victim}}^{\mathrm{train}}$ consisting of data samples *x*_*i*_, with their corresponding ground truth labels *y*_*i*_. The output of *f*_*victim*_(*x*) is a vector of *c*_*victim*_ dimensions, referred to as $\hat{y}$ . The goal of MIAs is to derive an attack model *f*_*attack*_(*a*), able to correctly estimate whether an arbitrary sample *x*_*new*_ was part of $D_{\mathrm{victim}}^{\mathrm{train}}$ , or not. The input of *f*_*attack*_(*a*) is the attack vector *a*, which can consist of arbitrary information available about the victim model, such as sample loss *L*_*x*_, the prediction vector ${\hat{y}}_{x}$ or metrics derived therefrom. In the case that no full access to *f*_*victim*_(*x*) is granted, the success of these attacks depends on the attacker’s ability to create a shadow model that mimics the behavior of the victim model and effectively learns the membership status of data points.

MIAs can differ drastically in the definition of the attack situation, i.e., in the degree to which information about the victim model is assumed to be available. Generally, MIAs can be divided into classifier-based and metric-based approaches. The original classifier-based MIA in Shokri et al. (2017) assumes no direct and unlimited access to *f*_*victim*_(*x*), therefore training several shadow models using similarly distributed shadow training sets to recreate the prediction behavior of the original victim model. An attacker model *f*_*attack*_(*a*) is then trained on the samples’ highest prediction confidences generated by the different shadow models, estimating whether the model was fed with a training or a test sample. This classifier-based MIA approach has been later simplified to work with a single shadow model and different data distribution by Salem et al. (2018), as well as working only with predicted labels instead of confidences (Choquette-Choo et al., 2021; Li and Zhang, 2021). Shokri et al. (2021) later also showed that explanations can be leveraged in the attack vector to further facilitate MIAs. Metric-based MIA approaches instead directly learn thresholds on metrics such as the maximum prediction entropy (Salem et al., 2018) or the loss (Yeom et al., 2018). Similar to MIAs, Attribute Inference Attacks (AIAs) can be used to infer further input attributes from a DL system (Duddu and Boutet, 2022).

### 2.2 Differential privacy

Differential Privacy (DP) is a mathematical framework that provides a probabilistic guarantee on the privacy protection of individuals in a dataset. In the context of machine learning, DP can be used as a private training paradigm to guarantee the privacy of individuals that were part of a model’s training dataset (Abadi et al., 2016). The method involves clipping the gradients, as well as the addition of a certain amount of randomly sampled noise before the weight update.

Let *D* be a database containing sensitive information about individuals, and let *M* be a randomized algorithm that takes *D* as input and produces an output in some set *R*. For any two databases *D* and *D*′ that differ in at most one row, and for any subset *S* of the output range *R*, the randomized algorithm *M* satisfies (*ϵ*, *δ*)-differential privacy if:

$$\mathrm{Pr}\left[M\left(D\right)\in S\right]\leq e^{\epsilon }\mathrm{Pr}\left[M\left(D^{\prime }\right)\in S\right]+\delta$$ (1)

Intuitively, this definition means that the probability of the algorithm outputting a particular result should not change much when a single individual’s data is added or removed from the database. The degree of privacy protection is controlled by *ϵ*, which directly influences the amount of noise added to the data. A smaller value of *ϵ* leads to higher privacy protection but may result in lower accuracy of the analysis results, while a larger value of *ϵ* provides higher accuracy but weaker privacy protection.

### 2.3 Explainable AI

By now, the topic of XAI covers a broad range of methods, including example-based explanations obtained by content-based image retrieval, counterfactual explanations through generative modeling or the disentanglement of the decision logic of complex deep neural networks. Human-centered explanation methods have a particularly high significance for the practical application of XAI, as they facilitate the interpretation of decision processes by the human stakeholder. Therefore, this work specifically compares the influence of the commonly used attribution-based XAI methods to the emerging field of concept-based XAI. For a more comprehensive overview of approaches in the domain of XAI, the reader is referred to Tjoa and Guan (2020).

#### 2.3.1 Attribution methods

Attribution methods are characterized by their ability to quantify the relevance of a particular input feature, or a feature group, to the decision process. These methods can be divided into gradient-based and perturbation-based methods. Simonyan et al. (2013) proposed the saliency mapping technique as one of the early gradient-based attribution methods. Here, the relevance of the input is defined as the gradient of the output, with respect to the input. Class Activation Maps (CAMs) proposed in Zhou et al. (2016) took advantage of the activation localization of the Global Average Pooling (GAP) layer by computing the weighted sum of the activation maps. To bypass limitations imposed by the need for GAP layers in CAM, GradCAM (Selvaraju et al., 2017) was proposed, weighting activation maps by the gradient of the output, with respect to the intermediate layers. Other variants of CAM were proposed, averaging the pixel-wise weights [GradCAM++ (Chattopadhay et al., 2018)] or using gradient information about the global contribution of the input instead [ScoreCAM (Wang et al., 2020)]. Integrated Gradient (Sundararajan et al., 2017) determines input relevance by transitioning the network’s input from a baseline image to the input sample of interest, while aggregating the gradients along the trajectory. While all of the above methods only allowed the quantification of positive, relevance, DeepLIFT (Shrikumar et al., 2017) allows both negative and positive influence quantification. By comparing the actual image gradients to a reference output, DeepLIFT allows the quantification of relevance even when the actual gradients are zero.

Perturbation-based attribution techniques focus on the iterative manipulation of the input sample to derive its importance. The Occlusion (Zeiler and Fergus, 2014) method, for example, involves the masking of the input image through a sliding window baseline patch. Differences in prediction confidence when feeding different occlusions are used to aggregate the information in the final attribution map. The main advantage of such model-agnostic methods is that they can be applied irrespective of the chosen architecture of the model. Inspired by this, Fong et al. (2019) proposed an optimization finding the occlusion of the most relevant input region, and Petsiuk et al. (2018) determined the input relevance by iterative random masking. An alternative perturbation-based approach called LIME (Ribeiro et al., 2016) uses occluded inputs and their corresponding model predictions to linearly approximate the local neighborhood of the complex deep learning model.

#### 2.3.2 Concept-based explainability

The output of an XAI method is commonly used as a means to facilitate the understanding of the decision-making process for a stakeholder. To foster such understanding of the decision-making process, the stakeholder is required to assign meaning to the explanations through the interpretation of the facts at hand (Palacio et al., 2021). Most common XAI methods provide explanans in the form of complex mathematical relationships (i.e., variants of the gradients, linear classifier weights, etc.) which complicate the interpretation and leave much room for error. Concept-based explanation methods tackle this problem by providing explanans in the form of facts about the decision-making which relate to abstract, human-defined concepts. Thus, reducing the margin for error in the interpretation process.

First concept-based XAI methods have been introduced in Kim et al. (2018) and Zhou et al. (2018). Zhou et al. (2018) propose to decompose the weight vector that translates the second last layer’s activation to the logit of a particular class. This is achieved by solving an optimization problem, constraining the decomposed weights to be both non-negative and sparse, for better interpretability. Testing with Concept Activation Vectors (TCAV) has been proposed by Kim et al. (2018). Concept Activation Vectors (CAVs) can be computed on any intermediate model layer through the training of linear binary classifiers separating the activations of concept examples from non-concept examples. The CAVs are defined as the normal to the hyperplane of the learned classifier. For the training of CAVs, positive and negative concept examples have to be collected. These CAVs can then be used to quantify the importance of a concept to the prediction of a particular class with the TCAV scores. A TCAV score is the fraction of samples *x*_*k*_ with the class label *k* which increase their class score *y*_*k*_ when being moved infinitesimally into the direction of the CAV for a concept, therefore having positive directional derivatives *S*_*c*,*k*,*l*_(*x*). For a given class *k* and concept *c* with activations at layer *l* the TCAV score is given as:

$$TCAV_{Q_{c,k,l}}=\frac{\left|x\in X_{k}:S_{c,k,l}\left(x\right)>0\right|}{\left|X_{k}\right|},$$ (2)

In addition to the quantitative concept analysis, Lucieri et al. (2020b) proposed the visual evaluation of concepts as Concept Localization Maps (CLMs). With g-CLM and p-CLM, they propose both gradient-based and perturbation-based ways to localize a particular concept *c* on the input image. An overview of other concept-based explanation methods beyond feature attribution is found in Yeh et al. (2021).

### 2.4 Related work

With the announcement of the General Data Protection Regulation (GDPR) (Council of the European Union, 2016), the first voices were raised proclaiming a conflict between privacy and explainability in machine learning (Grant and Wischik, 2020). Although privacy and explainability have widely been regarded as separate fields in previous research, some works already investigated their interdependence when combined in a single AI system. Milli et al. (2019) were the first to show that gradient-based explanations can be exploited to facilitate the extraction of models from prediction APIs. The authors show that the exposure of gradient-based explanations from models trained on MNIST and CIFAR-10 can decrease the number of queries required to reconstruct a model by a factor of 1,000 as compared to using the model outputs alone. Aïvodji et al. (2020) showed that also counterfactual explanations in tabular data can be exploited for model extraction attacks. The experiments indicate that diverse and high-fidelity explanations lead to higher vulnerability. Shokri et al. (2021) performed an extensive evaluation on the impact of different attribution methods on the vulnerability of the AI system to membership inference attacks. They found that in some cases the variance of explanation vectors suffices to yield good MIA accuracies. Moreover, backpropagation-based XAI methods were found to leak more information about data points as compared to perturbation-based methods. Duddu and Boutet (2022) extended previous work by investigating the impact of gradient- and perturbation-based model explanations on the success of Attribution Inference Attacks (AIAs) for tabular data. The results strongly suggested that the availability of explanations leads to higher vulnerability against AIAs as compared to using only model predictions.

Case-based explanations have the advantage of producing intuitive and easy-to-understand explanations based on images that are similar to the model input. However, these types of explanations can be critical in applications where the training data encompasses personal identities that cannot be exposed to the model users. Montenegro et al. (2021a) propose a generative model to privatize case-based explanations, as well as a way to derive counterfactual explanations. However, the authors later applied the method to glaucoma detection, revealing several drawbacks for the application in medical practice (Montenegro et al., 2021b; 2022).

Another direction of work deals with the privatization or anonymization of the input data before training of DL models. Recently, Gaudio et al. (2023) proposed an end-to-end ante-hoc model which allows privacy-preserving image compression. These compressed images can be used for classification and *post hoc* explainability analysis. However, the exact privacy vulnerability of such approaches in practice is still questionable.

Most previous work focused on the influence of explanations on the privacy of prediction models. However, recent work suggests that methods for privacy-preserving machine learning also have an influence on the quality of explanations. While Franco et al. (2021) were among the first to combine privacy and explanation methods, Saifullah et al. (2022) and Bozorgpanah et al. (2022) were the first to provide extensive analyses on the impact of privacy-preserving methods on XAI methods. Saifullah et al. (2022) investigated the impact of different privacy-preserving methods on attribution-based explanations in different domains including time-series, document image, and medical image analysis. Their study suggests that different privacy methods have different effects on the quality of attribution-based explanations, and that perturbation-based XAI methods are less affected by noise introduced through differential privacy. The work by Bozorgpanah et al. (2022) investigated the impact of privacy masking on shapley values in the domain of tabular datasets. The authors find that privacy and explainability are compatible in simple application scenarios, under limited conditions.

The increasing complexity of deep learning models and biomedical applications using AI raises high demands on the quality and ease-of-interpretability of XAI methods. However, particularly sensitive domains like medicine pose growing requirements to data protection and privacy. Previous research focused mostly on the effect of widely used attribution methods on privacy, but neglected the impact of highly relevant human-centered explanation methods. To the best of our knowledge, this is the first work to investigate the effect of concept-based explanations on the vulnerability of models towards privacy attacks such as MIAs.

### 2.5 Datasets

Concept-based explanation methods usually require a sufficiently large dataset of representative samples with expert-curated concept annotations. This poses a significant limitation to the applicable range of public datasets. This work chose two exemplar use cases of AI in biomedical image analysis, namely skin lesion analysis, and diabetic retinopathy classification. Moreover, a synthetic data use case, inspired by the problem of skin lesion analysis, is used to demonstrate the effects of concept-based explanations on the privacy of AI models. The datasets used for classification and concept learning in the respective use cases are presented in this section.

#### 2.5.1 Skin lesion analysis

The International Skin Imaging Collaboration (ISIC) hosts annual challenges on curated skin image datasets. Moreover, they provide the largest publicly accessible library of digital skin images^1^. In this work, the *ISIC* dataset is used as a fusion of all previously released challenge datasets cleaned from duplicates according to the recommendations in Cassidy et al. (2022). The dataset is used to train models in an 8-class classification task, discriminating between Melanoma (*MEL*), Nevus (*NV*), Basal Cell Carcinoma (*BCC*), Actinic Keratosis (*AK*), Benign Keratotic Lesion (*BKL*), Dermatofibroma (*DF*), Vascular Lesion (*VASC*) or Squamous Cell Carcinoma (*SCC*). The data is randomly split into a training (23,868), validation (2,653), and test (2,947) portion, while stratifying for the classification ground truth.

As *ISIC* does not contain enough concept annotations, *Derm7pt* is used for concept learning. The seven-point checklist criteria dataset (*Derm7pt*)^2^ proposed in Kawahara et al. (2018) consists of clinical and dermoscopic images of 1,011 skin lesions with extensive diagnosis and concept annotation. Each sample is labeled as either Basal Cell Carcinoma (*BCC*), Nevus (*NV*), Melanoma (*MEL*), Seborrheic Keratosis (*SK*), or a Miscellaneous class (*MISC*). Concept annotations include information about Blue-Whitish Veil, Dots & Globules, Pigment Network, Regression Structures, Streaks, and Vascular Structures. For pre-training of DP models, Derm7pt is split into training (413), validation (203), and test (395) portions, used in a 5-class classification task.

#### 2.5.2 Diabetic retinopathy detection

The telemedicine provider EyePACS provides one of the largest publicly available fundus image datasets. The dataset (*EyePACS*)^3^ consists of fundus images labelled by experienced clinicians for the presence of diabetic retinopathy (DR) on a scale from 0 to 4, according to the Early Treatment of Diabetic Retinopathy Study (ETDRS) scale. For each patient, images have been captured from the left and right eye. The complete dataset is split randomly into training (15,758), validation (7,763), and test (11,587) portions. Splitting is performed in a stratified manner, ensuring that any samples stemming from the same patient ID are only used together in one of the data portions. In this work, we followed the common procedure of performing binary DR classification, considering only stages 0 (no DR) and 1 (mild DR) as healthy images.

For concept learning, data from the STructured Analysis of the Retina (STARE) project is used^4^. The *STARE* database is a collection of 400 fundus images with extensive diagnostic labels, and over 40 expert annotations of diagnostic features visible on the images. In a pre-processing step, single manifestations are pooled into nine distinct concept classes, such that each concept contains a minimum of 45 positive examples. The resulting concepts are A-V Change, Artery Diameter, BV Specular Reflex, CNV Manifestation, Cotton-Wool Spot, Drusen, Hemmorhage, Retinal, or Subretinal Exudate, and Vein Diameter.

In addition to *EyePACS* and *STARE*, a third DR data set is used for additional pre-training of DP models. The *APTOS2019* dataset^5^ is provided by the Asia Pacific Tele-Ophthalmology Society and contains 3,662 samples collected from different patients located in rural India. All images have been graded according to the International Clinical Diabetic Retinopathy Disease Severity Scale (ICDRSS). *APTOS2019* is randomly split into training (2,050), validation (513), and test (1,099) portions. Analog to the *EyePACS* dataset, *APTOS2019* is used for binary classification of DR in this paper.

#### 2.5.3 Synthetic concept classification

The *SCDB* ^6^ dataset (Lucieri et al., 2020b) is a synthetic dataset inspired by the complex problems of skin lesion analysis. Images are classified into one of two classes based on the combinations of shapes present in a base shape, depicting the skin lesion. The shapes can be overlapping and redundant, but classification evidence is sparse and localized. Along with the class label, each image is supplemented by shape annotation maps, serving as ground truth explanations. By using simple geometric shapes as human-understandable concepts, *SCDB* significantly facilitates the evaluation of explanations in XAI. The dataset is randomly split into training (4,800), validation (1,200), and test (1,500) portions. Moreover, an additional portion for concept learning of 6,000 samples is used.

### 2.6 Experimental setup

This work investigates the interplay between concept-based explanation methods, privacy-preserving machine learning, and privacy attacks. To build a solid basis for comparison, three classification tasks (skin lesion analysis, diabetic retinopathy detection, and synthetic geometry detection) are considered on three state-of-the-art model architectures.

#### 2.6.1 Models

Three different state-of-the-art model architectures were investigated in the experimentation to account for potential differences in their vulnerability, namely *ConvNeXt*, *NFNet*, and *ResNet-50*. All experiments on *ResNet-50* were conducted on an implementation of the model with Group Norm replacing the standard Batch Norm layers, to allow for a fair comparison with the deferentially private trained models. The PyTorch Image Models (timm)^7^ python package is used to obtain all models for training.

#### 2.6.2 Model training

Baseline models were trained using a Stochastic Gradient Descent (SGD) optimizer with a batch size of 128 samples and a momentum of 0.9. A small hyperparameter search is performed over suitable learning rates in the set *LR* = 0.1, 0.01, 0.001. Four data augmentation strategies of different intensity are evaluated for each classification problem, and the strategy performing best in the baseline setting is later adapted for DP training. All models are trained for 200 epochs with an early stopping threshold of 30 epochs on the validation loss, using a plateau learning rate scheduler with patience of 10 epochs. The final model is chosen based on the lowest validation loss during training. Due to the high class-imbalance in the skin lesion analysis and diabetic retinopathy detection tasks, weighted random sampling is used in the baseline training procedures.

In addition to the baseline model trainings, another set of models is trained to simulate the scenario of overfitting. To this end, trainings were performed without any weighted sampling, shuffling, learning rate scheduler or data augmentation (apart from normalization). Training is performed for 50 epochs without early stopping, and the last model instance is used for further experimentation.

For each classification task, the augmentation procedure performing best in the baseline setting is adapted for the corresponding DP trainings. The *Opacus* ^8^ python package is used for establishing the DP pipeline. DP trainings are manually tuned to achieve a good trade-off between test accuracy and (*ϵ*, *δ*)-privacy. Therefore, different batch sizes, learning rate schedulers and settings for *ϵ* and max _*grad*_*norm* have been used.

#### 2.6.3 Concept training and explanation

For each model, CAVs are trained following the procedure outlined in Lucieri et al. (2020a). 100 different CAVs are computed with different seeds for the random undersampling procedure, to ensure balanced concept sets. A final CAV is computed by computing the average direction over all CAVs. Concept activations are extracted from the last layer of the main network blocks for all architectures. In skin lesion analysis, *Derm7pt* is used for CAV computation. *STARE* is used as the concept dataset in diabetic retinopathy detection. *SCDB* comes with a specific dataset portion which is used for CAV computation.

All CLMs are computed according to the procedure outlined in Lucieri et al. (2020b). Both the gradient-based g-CLMs and the perturbation-based p-CLMs are considered in this study. Saliency attribution is used to produce the concept relevance in g-CLMs, whereas occlusion with a window size of 15, and a stride of 8 is used for p-CLMs. Occluded patches are filled with blurred patches attenuated by a circular Gaussian filter. For concept predictions, an aggregated concept prediction vector is assembled from all individual binary concept classifiers.

#### 2.6.4 Attribution computation

In this work, both gradient-based and perturbation-based attribution methods are compared. For gradient-based attribution the Saliency method is chosen, as it provides more sensitive information about the decision-making process, and has already shown to promote privacy leakage in some cases (Shokri et al., 2021).

The Captum ^9^ python package is used for the computation of all Occlusion and Saliency attribution maps. For Occlusion, window sizes and strides are chosen to match the parameters of the p-CLM computation. Occluded patches are filled by the mean over the individual image’s pixels.

#### 2.6.5 Membership inference attacks

In this paper, experiments are performed both on metric-based and classifier-based MIAs. The datasets used to perform and evaluate MIA attacks consist of equal parts of seen and unseen samples. Depending on the deployment scenario, the unseen data is taken from an identical (Optimal Deployment Scenario) or slightly different distribution (Suboptimal Deployment Scenario).

For prediction vectors, metric-based attacks are performed on the maximum prediction score as well as its entropy and variance. For attribution maps and CLMs, metric-based attacks are computed by using the variance over individual attribution maps or the individual set of CLMs. Metric-based attacks are evaluated on the whole MIA dataset.

Classifier-based MIAs are conducted using Support Vector Machines (SVMs) and Neural Networks (NNs). In both cases, the individual feature groups (i.e., attribution maps, CLMs, etc.) are scaled to 0 mean and unit variance before combining the final attack vector. SVM-based MIAs are trained and evaluated with a split ratio of 67/33. NN-based MIAs are conducted on a fully connected network consisting of six linear layers and ReLU inspired by Shokri et al. (2021). The corresponding attack vectors are flattened before being fed to the network. NN-based MIAs on attack vectors including attribution maps and CLMs have additionally been computed with *VGG16* and *ConvNeXt* architectures. Therefore, the whole attack vector is flattened, padded with zeros, and reshaped to a variable number of channels *c* to fit in a rectangular shape suitable for the corresponding network. All NN-based attack models are trained for a maximum of 100 epochs with the SGD optimizer, and an early stopping threshold of 10 epochs to ensure convergence of the models. A train, validation, and test split with a ratio of 45/22/33 is used.

**FIGURE 1:**
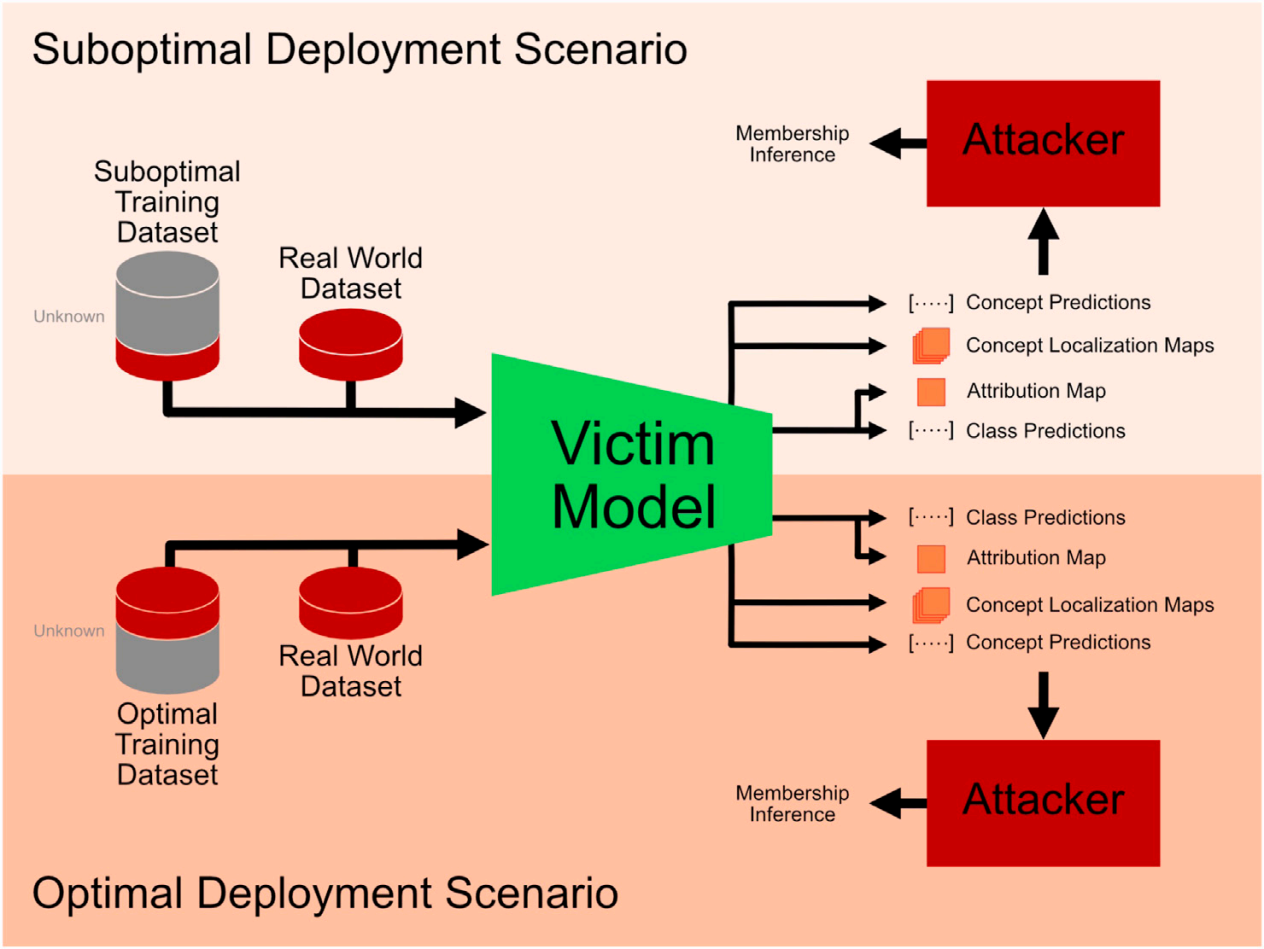
Overview of the two deployment scenarios considered for the membership inference attacks. In the suboptimal deployment scenario, the training dataset does not fully capture the data distribution of the real-world data. In the optimal deployment scenario, the training dataset and the real-world dataset are assumed to come from highly overlapping dataset distributions.

In all experiments, maximum access to *f*_*victim*_(*x*) is assumed, as the main aim of this investigation is to analyze the increase in privacy vulnerability due to the addition of concept-based and attribution-based explanations. This means that the adversary can obtain the attack vectors used in the MIAs directly from the victim model’s API. Therefore, the results reflect an upper bound on the expected privacy vulnerability. Moreover, two distinct scenarios are considered, as depicted in Figure 1. The first scenario, referred to as suboptimal deployment scenario, is meant to reflect an easier attack scenario that easily allows quantifying the differences in attack vulnerability of different attack vectors. The assumption here is that the network has been trained on a set of data with a narrow distribution, which is not fully representative of all realistic real-world data. The second scenario, referred to as the optimal deployment scenario, assumes that the training data distribution fully reflects the real-world data distribution. These two scenarios are simulated by varying the data the attacker has access to. In common biomedical classification problems like skin lesion analysis and diabetic retinopathy detection, large public reference databases (such as the *ISIC* or *EyePACS*) exist. Therefore, it is reasonable to assume that the adversary has access to at least a part of the training data stemming from these public repositories. In the suboptimal deployment setting, it is assumed that the distribution of the data used for training does not overlap significantly with the real-world data distribution. To simulate this, an unseen subset of data from a slightly different distribution, namely the test set of the *Derm7pt* dataset, is used as the data reflecting non-membership to the training data. In the optimal deployment setting, it is assumed that the distribution of the data used for training is mostly representative of the variations present in relevant real-world imaging. To simulate this, the unseen test split of the *ISIC* dataset is used as the data reflecting non-membership to the training data. In all MIA scenarios, training, validation and test data is balanced, to avoid misleading performance metrics.

## 3 Results

The following chapter presents the results from the previously described experiments. After outlining the performances of all trained classification models, the effect of concept-based explanations on model privacy is first theoretically investigated in the suboptimal deployment scenario when applying metric-, SVM-, and NN-based attacks. Afterward, the results from the optimal deployment scenario are described to quantify the effect in practice. Finally, the results for CAV training on DP-trained networks are presented to show the effect of privacy on concept-based explanations.

### 3.1 Model training results

More than 50 models were trained in the initial phase. The weighted average F1-scores of the final selected models, evaluated on the respective test datasets, are presented in Table 1. Models trained on the *SCDB* dataset performed best in the baseline setting. Over-fitting led to a slight decrease in performance, while models trained using the DP procedure performed significantly worse, with up to 14% lower F1-scores. Overall, *NFNet* achieved the best results for *SCDB* over all training settings. For the biomedical datasets, it can also be observed that *NFNet* usually led to slightly better results as compared to *ResNet50* and *ConvNeXt*. Both *ConvNeXt* and *NFNet* performed best in the baseline setting for *ISIC*, while *NFNet* performed better in the overfit, and on par in the DP setting. For *EyePACS*, *NFNet* achieved the best weighted average F1-scores over all settings.

**TABLE 1:** Weighted average F1-scores of all trained models on the respective test datasets. For models trained with Differential Privacy, the utilized privacy budget *ϵ* is given in brackets.

Dataset	Architecture	Baseline (%)	Overfit (%)	DP (%) (*ϵ*)
SCDB	ResNet50	97.79	92.33	83.74 (05)
	NFNet	98.26	93.93	85.29 (05)
	ConvNeXt	97.39	91.06	83.79 (05)
ISIC	ResNet50	84.50	77.75	70.00 (10)
	NFNet	88.14	84.70	68.34 (05)
	ConvNeXt	88.78	78.68	65.45 (05)
EyePACS	ResNet50	86.72	87.39	82.29 (05)
	NFNet	88.89	88.48	84.77 (10)
	ConvNeXt	86.89	87.82	81.69 (10)

**FIGURE 2:**
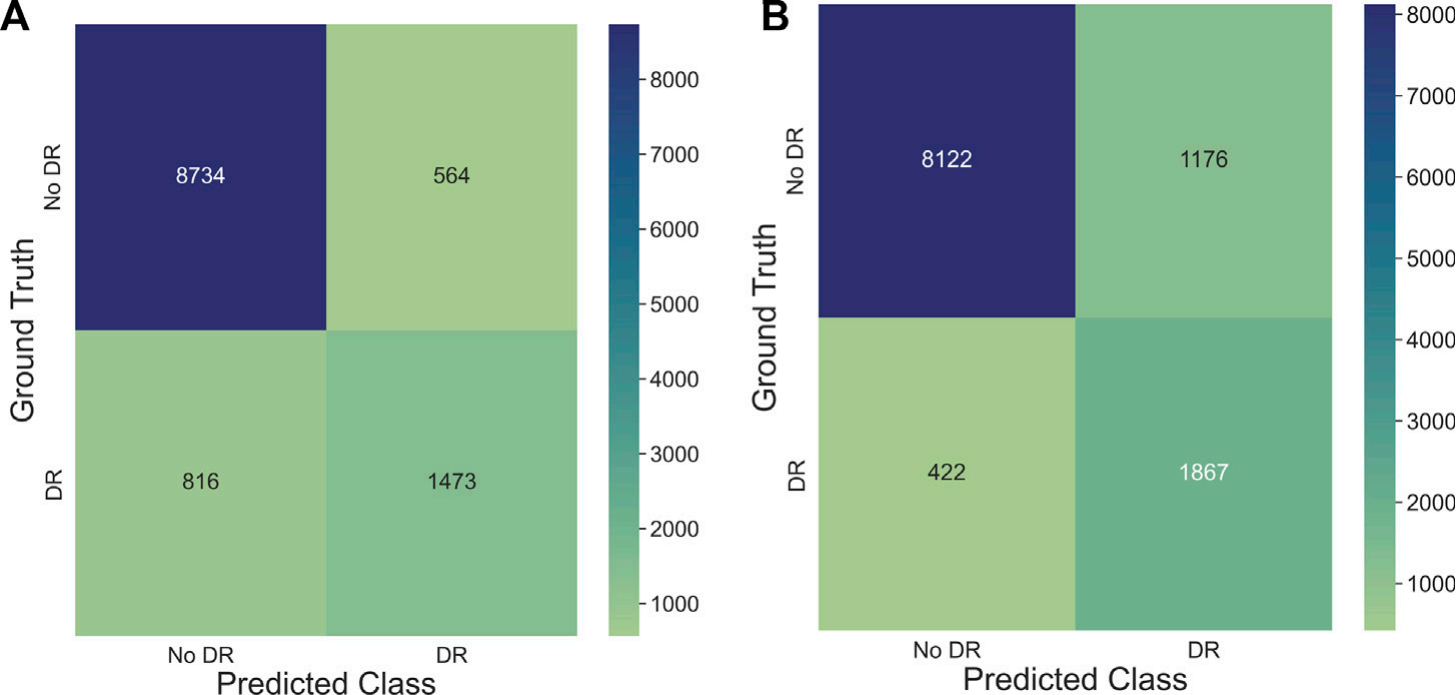
Confusion matrices generated on test set evaluation for *ConvNeXt* models trained on the *EyePACS* dataset with **(A)** baseline training setting optimized for performance and **(B)** training setting optimized for overfitting. It can be seen that the higher test accuracy achieved by the overfitted model is a result of its higher tendency to predict the majority class.

The training settings tailored for overfitting led to a noticeable decrease in F1-scores for *ISIC* and *SCDB* datasets (−6.8% and −5.4% on average, respectively). For *EyePACS*, on the other hand, there was no significant change in performance when overfitting the models. Two of the architectures scored even higher when trained without weighted sampling, augmentation and shuffling (0.7% and 0.9% for *ResNet50* and *ConvNeXt*, respectively). However, inspecting the confusion matrices of both baseline and overfitted *ConvNeXt* models in Figure 2, it can be clearly seen that the overfitted model simply developed a stronger tendency to predict the majority class, leading to higher F1-scores on the test set.

Training with DP led to a significant decrease in test F1-scores in the majority of the cases. The highest average decrease of −19.2% can be observed with the *ISIC* dataset, as it has a strong dataset imbalance among the 8 classes. Among both binary datasets, *SCDB* suffered more from DP training on average (−13.5% compared to −4.6%). This is most likely due to the previously reported tendency of *EyePACS* models to perform well when focusing mostly on the majority class.

In an attempt to decrease the performance gap between baseline and DP settings, the application of under sampling and class-weighted losses have been investigated for both imbalanced medical datasets. The oversampling setting is not investigated here, as it would contradict with the basic assumption of DP that every forward pass with a certain sample instance reduces the privacy budget of that instance. As can be seen from Table 2, undersampling always led to a significant decrease in test F1-scores (−12.33% and −4.96% for *ISIC* and *EyePACS*, respectively). The use of a class-weighted loss, on the other hand, led to a slight increase in test F1-score in the case of *EyePACS*, while reducing the score for *ISIC* by −2.24%. An inspection of the test confusion matrix, however, reveals that only in the case of undersampling, the DP trained model can predict all eight individual classes for *ISIC*. For both non-weighted and class-weighted loss, the DP models focus heavily on the majority classes.

**TABLE 2:** Weighted average F1-scores of *ResNet50* models trained on *ISIC* and *EyePACS* with differential privacy (*ϵ* = 10) under different imbalanced training settings.

Dataset	ISIC (%)	EyePACS (%)
Baseline	66.81	82.56
Weighted Loss	64.57	82.76
Undersampling	54.48	77.60

### 3.2 The effect of concept-based explanations on model privacy in theory

This section reports the results of different MIAs considering their theoretical impact in a suboptimal deployment scenario to outline the vulnerability of different explanation variations. MIAs are applied to *ISIC* and *EyePACS* models exposing no explanations, attribution-based explanations, or concept-based explanations to compare and quantify the impact of different explanation levels on the vulnerability of networks. First, the results of metric-based attacks are reported, followed by SVM-based and NN-based approaches.

#### 3.2.1 Metric-based attacks

**FIGURE 3:**
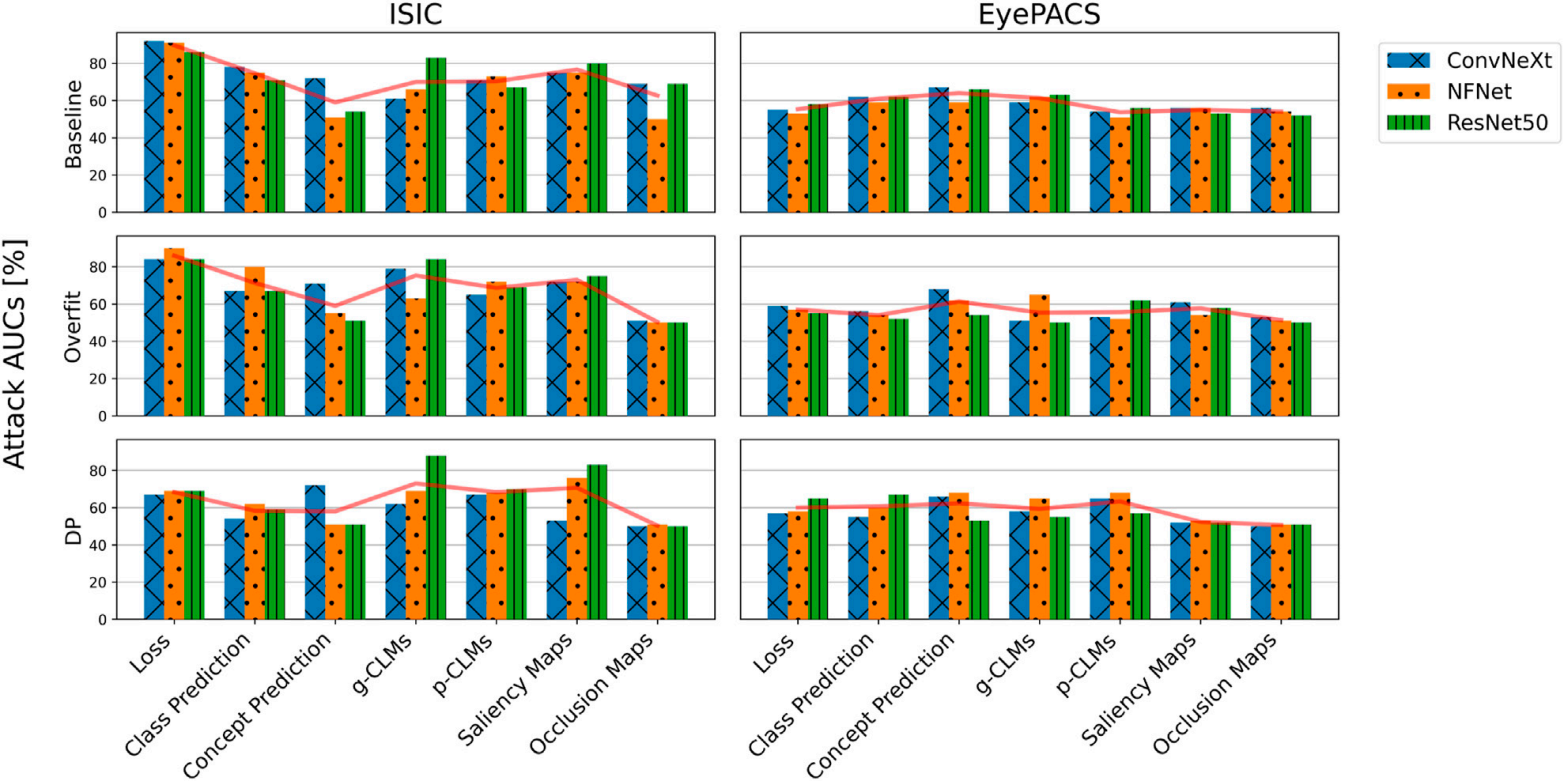
AUCs of metric-based membership inference attacks applied on all *ISIC* and *EyePACS* models in the suboptimal deployment setting. The average AUC over different model architectures is indicated by the red lineplot.

Figure 3 shows the AUCs of different metric-based attacks on all trained models. For the *ISIC* dataset, the loss is clearly the most impactful variable to be used in an MIA, resulting in AUCs of up to 92%, followed by saliency attribution maps and class predictions with an average AUC in the baseline of 77% and 75%, respectively. Both CLM versions and gradient-based attribution maps resulted in comparable performances. Occlusion, however, yielded lower attack AUCs on average. The worst average AUC is reported by concept predictions, with an average of 59% in the baseline settings. Surprisingly, the results for the *EyePACS* dataset suggest that concept predictions are most informative for MIAs, while attribution maps result in the lowest AUCs. The loss, however, performed only slightly above chance with an average AUC of 55%. Moreover, both gradient-based and perturbation-based attribution maps yielded lower AUCs for *EyePACS*, while g-CLMs led to 5.5% higher AUCs on average. Although general trends are observable, AUCs can vary significantly between different model architectures. Over the baseline results for both *ISIC* and *EyePACS* it can be observed that both *ConvNeXt* and *ResNet50* are, on average, more vulnerable as compared to *NFNet*, with average AUCs of 66%, 66%, and 63%, respectively.

The trends reported for the baseline training setting are similarly reflected in the other two training settings. For both datasets, a peak in concept prediction vulnerability in the overfit setting is noticeable. Moreover, occlusion attribution seems to drastically lose information value in the overfit and DP settings. Overall, it can be noted that the highest attack AUCs are achieved on baseline models, followed by overfit and DP, as backed by the average AUCs of 65%, 63%, and 61%, respectively. However, AUCs of different metrics suffer to a different extent from overfitted or DP training. While metrics like the loss, or attributions suffer higher AUC losses in DP, metrics based on concept-based explanations remain the same or sometimes even increase (e.g., p-CLMs on *EyePACS*).

#### 3.2.2 SVM-based attacks

**FIGURE 4:**
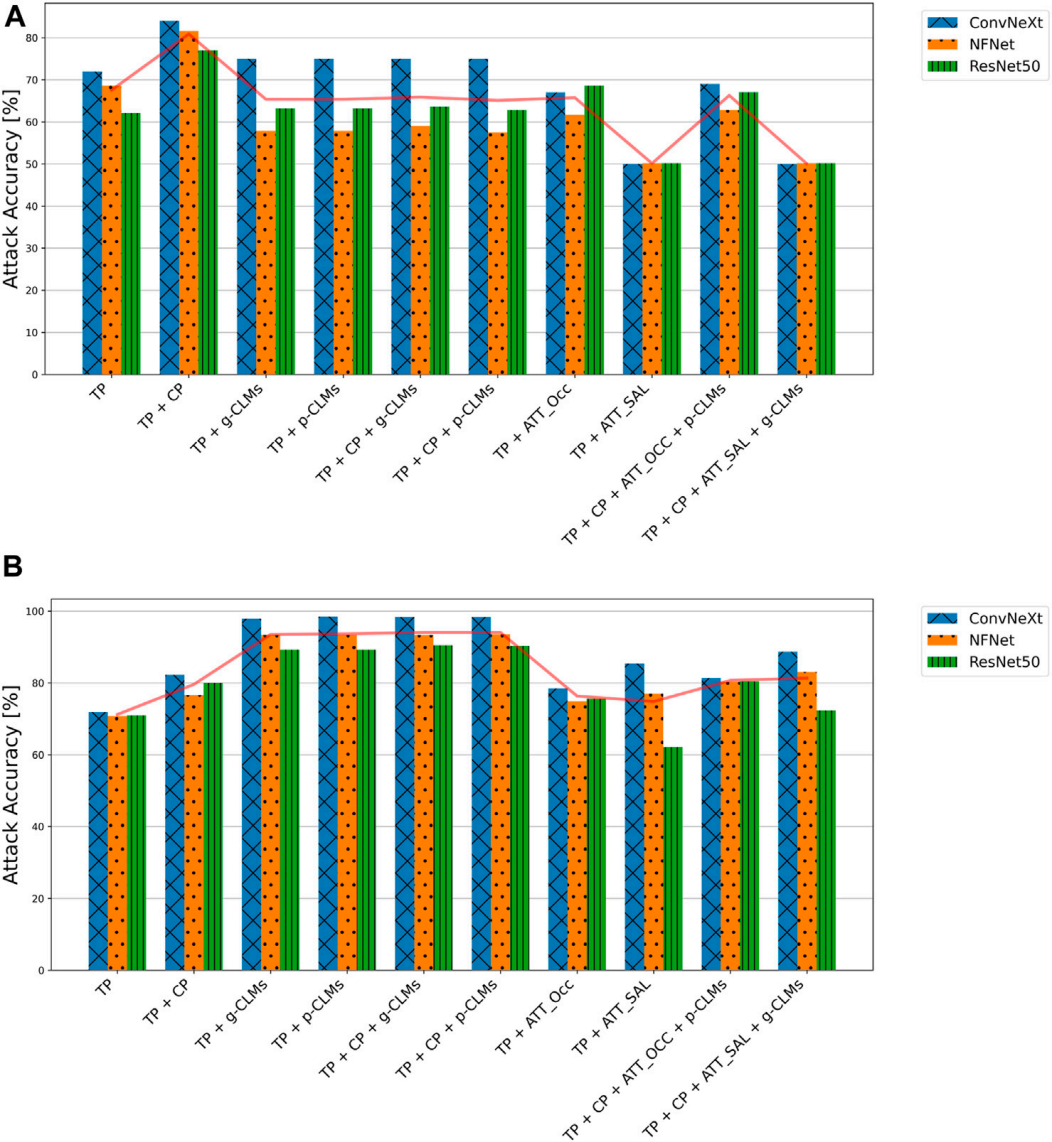
Attack accuracies of SVM-based membership inference attacks in the suboptimal deployment setting for all model architectures trained on **(A)** *ISIC* and **(B)** *EyePACS* datasets for ease of legibility, the components of the attack vectors have been shortened as *TP* for target class prediction, *CP* for concept prediction, *ATT_Occ* for Occlusion attribution maps, and *ATT_SAL* for Saliency attribution maps.

Figure 4 shows the test accuracies of SVM-based attacks on baseline models from different architectures, using different attack metrics for the *ISIC* and *EyePACS* datasets. For the *ISIC* dataset, it can be observed that *ConvNeXt* was most vulnerable, on average, whereas features from *NFNet* and *ResNet50* resulted in lower attack accuracies. It is also clear from the results that concept predictions significantly increase the attack accuracies as compared to the normal target class predictions. Interestingly, all high-dimensional attack vectors led to lower attack accuracies in the SVM-based attacks. Moreover, attacks including Saliency attribution maps were not conclusive at all.

The results for *EyePACS* draw a similar picture. The attack models achieve an average accuracy of 71% using only class predictions as the attack vector. Adding concept predictions or CLMs to the attack vector increases the average vulnerability by 9% or 24%, respectively. Interestingly, there is only a minor difference between results for gradient-based and perturbation-based CLMs. Moreover, adding concept predictions to the attack vector composed of class predictions and CLMs leads to marginal improvement. Attack vectors composed of class predictions and attribution maps tend to yield lower attack accuracies. Whereas the perturbation-based attribution yielded largely consistent results, the performance of attacks based on gradient-based occlusion varied depending on the underlying architecture of the victim classifier. Overall, the results indicate a higher average vulnerability of *ConvNeXt* as compared to *NFNet* and *ResNet50*. Similar to *ISIC*, all attack vectors that include attribution maps yielded lower attack accuracies as compared to the attacks based on concept-based explanations only.

#### 3.2.3 NN-based attacks

**FIGURE 5:**
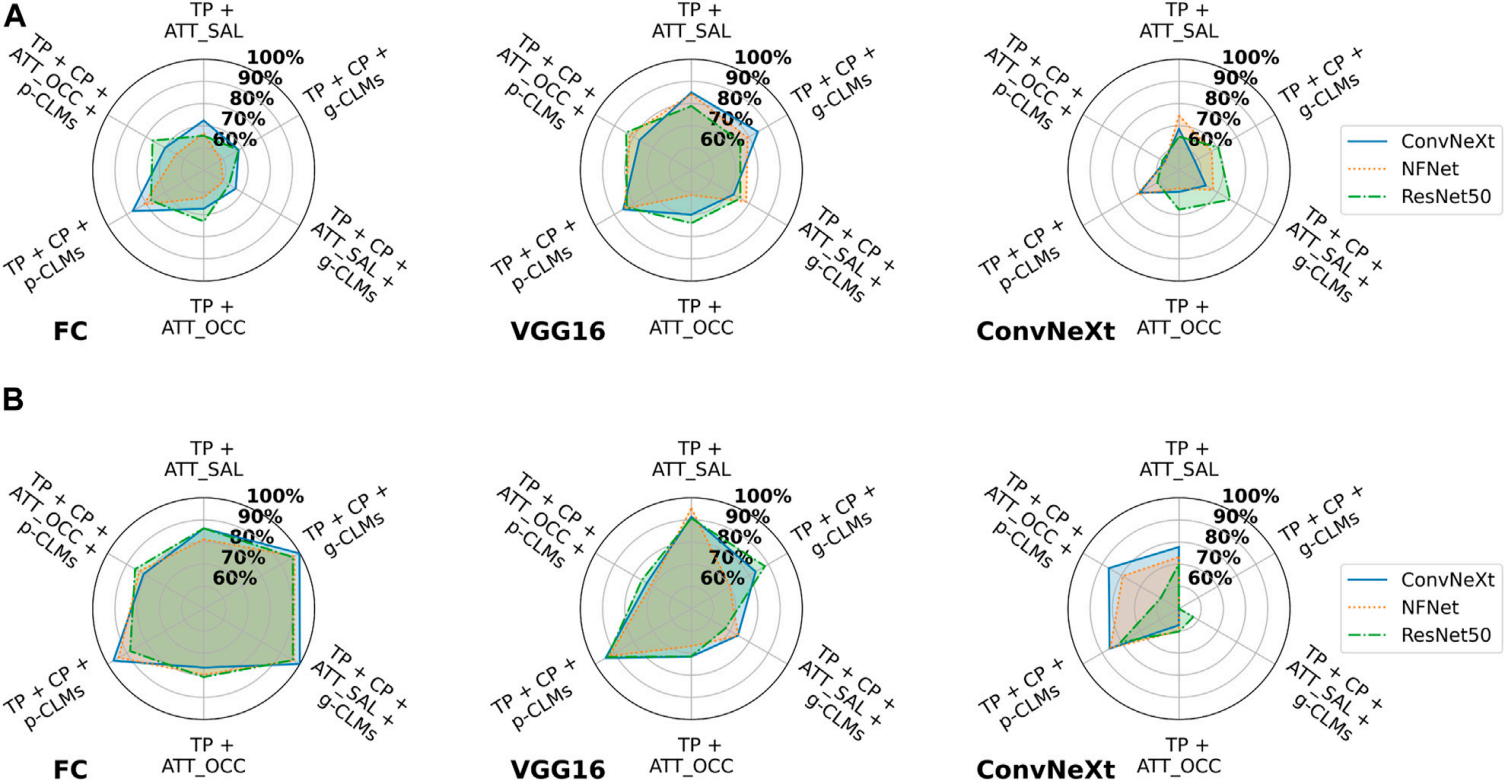
Attack accuracies of NN-based membership inference attacks in the suboptimal deployment setting for all model architectures trained on **(A)** *ISIC* and **(B)** *EyePACS* datasets with three different attack architectures. For ease of legibility, the components of the attack vectors have been shortened as *TP* for target class prediction, *CP* for concept prediction, *ATT_Occ* for Occlusion attribution maps, and *ATT_SAL* for Saliency attribution maps.

The attack accuracies from NN-based attacks are presented in Figure 5 for both datasets and varying attack architectures. First, it can be observed that *VGG16* and *FC* usually resulted in the best attack accuracies, while *ConvNeXt* performed worst. Moreover, results from *FC* and *VGG16* attack models indicate that *ConvNeXt* victim models are slightly more vulnerable as compared to *NFNet* and *ResNet50*.

Overall, the experiments indicate that the combination of the target class prediction vector, concept predictions and perturbation-based CLMs (*TP + CP + p-CLMs*), results in the highest vulnerability for most attack cases. For *ISIC*, any attack vector combination with gradient-based CLMs surprisingly yielded lower attack accuracies. In the case of *VGG16*, Saliency attribution maps performed comparable to the attacks based on concept-predictions. Attack vectors combining all types of explanations typically result in lower vulnerability as compared to attack vectors based only on concept-based explanations, except for *FC* for *EyePACS*.

**FIGURE 6:**
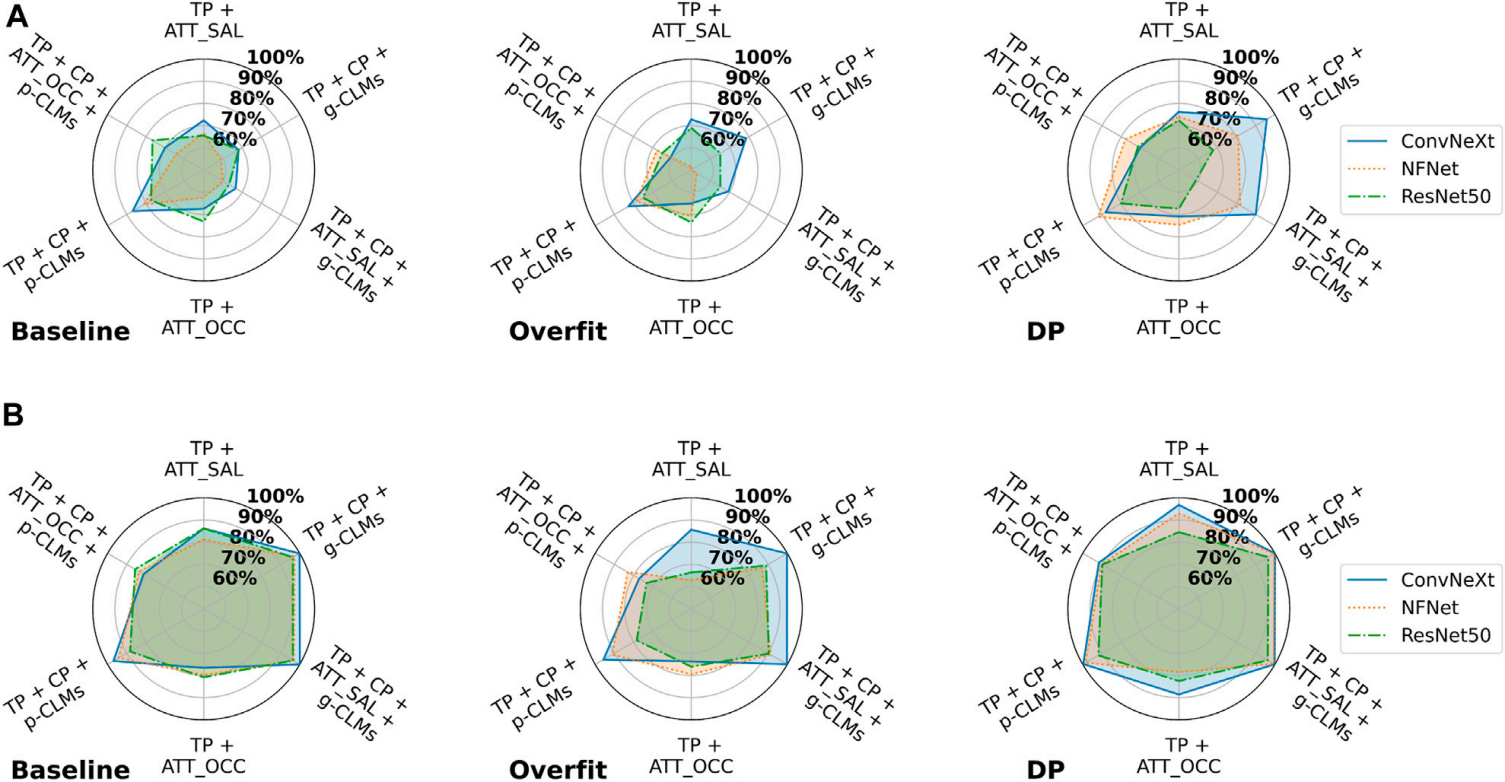
Attack accuracies of NN-based membership inference attacks using a fully connected network on all models in the suboptimal deployment setting for all model architectures trained and training strategies on **(A)** *ISIC* and **(B)** *EyePACS* datasets. For ease of legibility, the components of the attack vectors have been shortened as *TP* for target class prediction, *CP* for concept prediction, *ATT_Occ* for Occlusion attribution maps, and *ATT_SAL* for Saliency attribution maps.

Figure 6 shows the effect of different training settings on the attack accuracy when using the FC attack architecture. It is strikingly visible that for both datasets, DP leads to an increase in vulnerability for all models. This effect is most strongly apparent in attack vectors including concept-based explanations. However, in the case of *EyePACS*, attribution-based attack accuracies increase for DP as well. The overfit training setting, on the other hand, shows several cases where the attack accuracy decreases as compared to the baseline.

### 3.3 The effects of concept-based explanations on model privacy in practice

This section presents the results of MIA attacks in the more challenging, optimal deployment scenario, where the training dataset distribution has a significant overlap with the real-world data.

**FIGURE 7:**
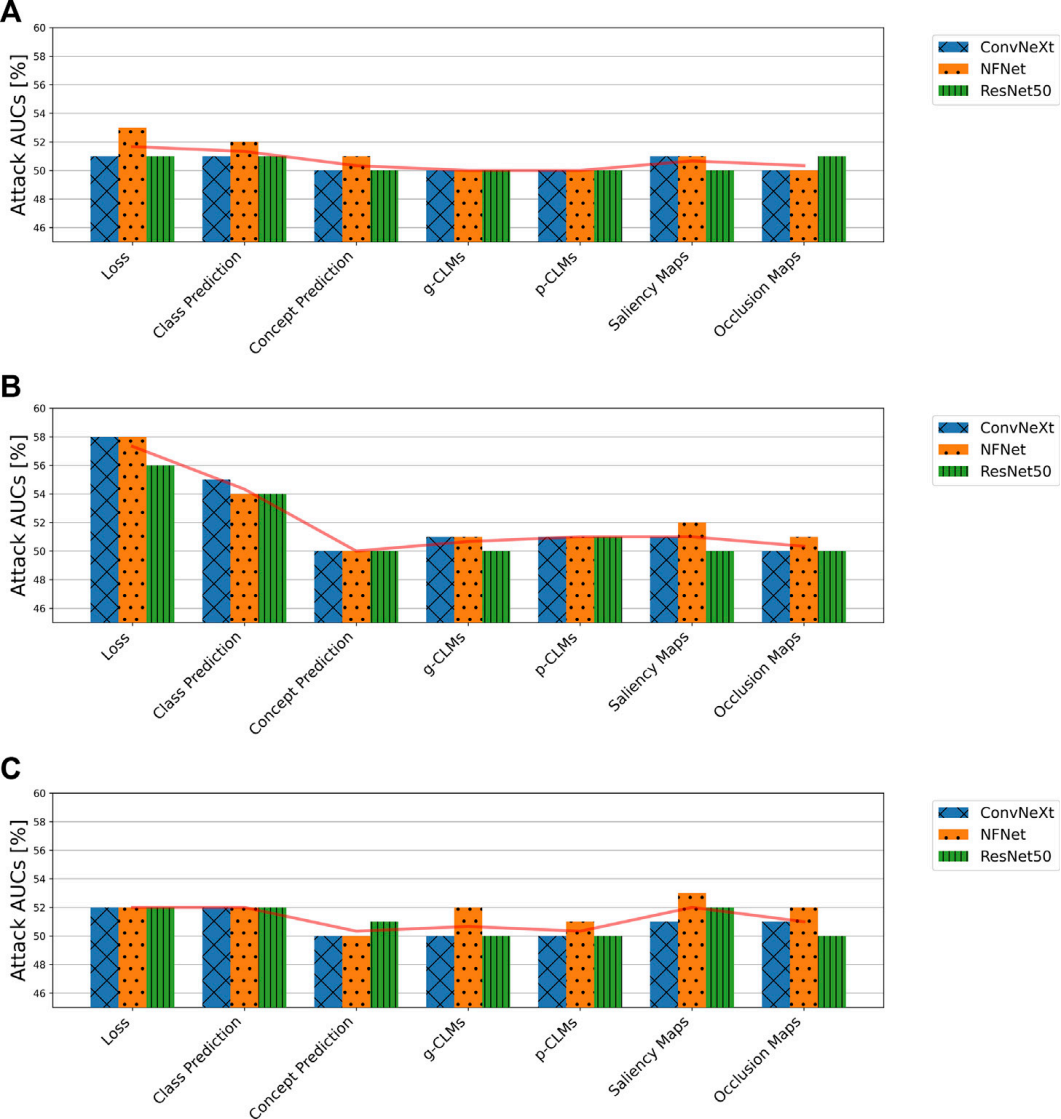
Attack accuracies of metric-based membership inference attacks in the suboptimal deployment scenario for all model architectures trained on **(A)** *SCDB*, **(B)** *ISIC*, and **(C)** *EyePACS* datasets.

Figure 7 shows the attack AUCs of metric-based attacks on the *SCDB*, *ISIC*, and *EyePACS* datasets. It can be observed that all attack AUCs for all datasets lie below 60%. For *EyePACS* and *SCDB*, AUCs even lie below 53% with no consistent behavior between different attack metrics. Therefore, the attacks can be considered as unsuccessful. However, for the *ISIC* dataset it can be seen that the loss consistently yields the highest attack AUCs, followed by the class predictions.

**FIGURE 8:**
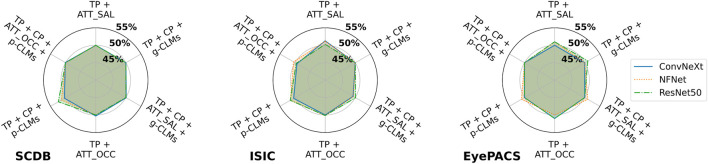
Attack accuracies of NN-based membership inference attacks using the *FC* attack network on *SCDB*, *ISIC*, and *EyePACS* models in the suboptimal deployment scenario. For ease of legibility, the components of the attack vectors have been shortened as *TP* for target class prediction, *CP* for concept prediction, *ATT_Occ* for Occlusion attribution maps, and *ATT_SAL* for Saliency attribution maps.

The same picture is drawn by the SVM-based and NN-based attacks. Figure 8 shows an example of classifier-based attacks using the *FC* attack network on *SCDB*, *ISIC*, and *EyePACS*. It can be seen that barely any membership inference is possible in this scenario. Moreover, no significant trend or advantage is observable for a specific attack vector combination.

### 3.4 The effects of DP on CAVs

Table 3 shows the mean and standard deviations of test accuracies for CAVs evaluated on the test portion of the concept datasets. Results are averaged over all model architectures for each of the training settings. In addition to the dataset-wise observation, the average over all datasets is presented in the last column. The results for the synthetic *SCDB* dataset clearly show that there is a strong correlation between the model performance and the corresponding CAV accuracy. With decreasing model performance from baseline to DP settings, the CAV accuracy decreases as well, while the standard deviation of the results increases significantly (from ± 1.83 in baseline to ± 2.68 in DP). This overall trend is confirmed when inspecting the results for the *EyePACS* dataset, as well as the aggregated statistics over all datasets. The *ISIC* dataset, however, draws a slightly different picture in the average accuracy, with a minor increase in concept accuracy for the DP setting, while the lowest average concept accuracy is reported in the overfitted training setting. The standard deviation, on the other hand, is highest in the DP setting, whereas the overfitted setting shows the lowest value. However, these results are surprising considering the fact that all architectures trained on *ISIC* suffered the highest relative drop in performance from baseline to DP settings.

**TABLE 3:** Mean and standard deviation of the CAV test accuracies over all model architectures tested on the concept data test split. Results are given averaged over all datasets, as well as the averages over model architectures trained on single datasets.

Dataset	SCDB		ISIC		EyePACS		Overall	
	Mean	Std	Mean	Std	Mean	Std	Mean	Std
**Baseline**	84.03%	±1.83	68.93%	±3.99	64.41%	±6.99	72.46%	±4.27
**Overfit**	77.89%	±2.26	67.80%	±3.46	62.72%	±7.01	69.47%	±4.25
**DP**	73.69%	±2.68	68.13%	±4.31	61.58%	±7.06	67.80%	±4.68

The average cosine similarities between the 100 individually computed CAVs are given in Table 4, averaged over all architectures in different training settings. In addition to the dataset-wise observation, the average over all datasets is again presented. A cosine similarity of 1 indicates the total alignment of vectors, whereas 0 indicates orthogonality. The results for the synthetic *SCDB* dataset indicate that the CAVs are more aligned in the baseline settings, while CAVs in overfit and DP settings tend to deviate more from one another. For *ISIC* and *EyePACS*, results show only slight to no significant differences in cosine similarity of CAVs. However, in both cases overfit resulted in the lowest cosine similarity, while DP resulted in the highest values. Overall, it can be seen that the baseline setting leads to the highest alignment of CAVs, followed by DP and overfit.

**TABLE 4:** Cosine similarities of individual CAVs trained on concept data. Results are given averaged over all datasets, as well as the averages over model architectures trained on single datasets. Cosine similarity of 1 indicates full alignment, while 0 indicates orthogonality.

Dataset	SCDB	ISIC	EyePACS	Overall
Baseline	0.44	0.46	0.40	0.44
Overfit	0.37	0.44	0.38	0.40
DP	0.35	0.48	0.42	0.42

## 4 Discussion

Concept-based explanations are a particularly useful tool for building human-aligned machine interfaces. In contrast to traditional explanation methods, they specifically utilize the same high-level concepts known to their relevant stakeholders in their explanation. This drastically improves the intelligibility of the system, and therefore facilitates the integration of AI in complex real-world use cases. However, making the decision behavior more transparent should not compromise a model’s robustness against attacks by adversaries. Particularly in healthcare and biomedicine, membership inference attacks harbor the risk of disclosing sensitive information about data subjects. The results presented in Section 3 indicate some interesting interactions between concept-based explanations and the vulnerability of victim models under membership inference attack. In the following, some main findings are presented and discussed in more detail.

### 4.1 Class-imbalance is one of the biggest problems in private biomedical image analysis

The results of the model trainings indicate that *NFNet* has the most robust performance over datasets and training settings. However, *ConvNeXt* and *ResNet50* performed comparably in many settings. The F1-scores reported for models trained on *SCDB* align with the initial expectations of lower performance when overfitting, and significantly lower performance when training with differential privacy. It is striking, however, that even for the well-balanced *SCDB* dataset, DP led to two-digit performance drops.

Models trained on the *ISIC* dataset suffered substantial performance drops from overfitting and DP training. This is reasonable when considering that it is an 8-class classification problem with high dataset imbalance. The high imbalance with some minority classes having no more than 194 instances in the training set also leads to a drastic reduction in training set size when under sampling. However, under sampling also failed to improve or at least maintain the model performance in the case of the much more balanced *EyePACS* dataset with 6,138 total samples left in the undersampled training dataset. Moreover, class-weighted losses have had a very insignificant impact on imbalanced training, while even decreasing the model performance in problems with very high class imbalance. Interestingly, models trained on the *EyePACS* dataset showed less performance drops when overfitting and training using the DP procedure. This is partly because *EyePACS* is formulated as a binary problem. As seen in the confusion matrices, it can also be assumed that the detection of diabetic retinopathy is a sufficiently complex problem, where even baseline models suffer from a significant amount of overfitting to the original data distribution.

From a practical perspective, the results strongly highlight the importance of class-balancing in biomedical imaging. It is in the nature of the healthcare system, that biomedical imaging workflows are strongly outcome and context biased. This means that some imaging procedures are usually undertaking only in very severe and suspicious cases. Therefore, the quantity of documented disease can vary widely depending on the clinical relevance, leading to high class imbalances. Moreover, examinations in certain facilities often correlate highly with extremely malignant, or exceptional cases of a disease. All of these factors lead to implicit biases and spurious correlations in datasets, which data-driven algorithms can easily pick up. This is often very hard to reveal without highly curated gold standard evaluation datasets. As shown in this study, class imbalances in biomedical datasets are even more important in cases where privacy is of interest. This is also in line with previous findings on the severe effect of data imbalance on differential privacy (Farrand et al., 2020). On one hand, researchers should put more efforts into the development of training methods that allow good learning behavior in cases of imbalanced datasets in normal and differentially private training settings. Ultimately, it should be the goal to curate more high-quality datasets in biomedical domains, which are broad enough and representative of many case variations.

### 4.2 Concept-based explanations could increase the vulnerability of private models

The comparison of attack vulnerabilities in the suboptimal deployment scenario gives valuable insight into the impact of concept-based explanations to the model privacy. Results from the metric-based attacks suggest that concept-based explanations can sometimes lead to higher model vulnerability as compared to classical attribution maps. In the case of *EyePACS*, the attacks on concept predictions were even more successful as compared to using the loss as the attack vector. SVM-based attack results confirm these findings, highlighting that the impact of concept predictions depends on the training dataset. This might also correlate with CAV accuracy, as concept-predictions led to less vulnerability in *EyePACS*, which presented lower average CAV performance. Both SVM- and NN-based attacks support the finding that concept-based explanations clearly outperform attribution methods as attack vectors for MIAs. Moreover, the results indicate that when concept-based explanations are present, the addition of attribution maps to the attack vector is usually deteriorating attack accuracies. This might stem from the fact that the drastic increase in attack vector dimensionality complicates the extraction of relevant features, even for NN-based attack models.

The theoretical investigation indeed revealed that concept-based explanations can potentially increase the vulnerability of systems, under some conditions. One pragmatic intuition that could justify why concept-based explanations lead to a higher vulnerability, is the sheer fact that CLMs of different CAVs expose a higher dimensional internal representation of the network, as compared to the single attribution map that is typically presented only for the class predicted by the network. However, it has also been shown that in some cases the concept prediction vector alone can yield higher attack accuracies as compared to the CLMs or attribution maps. This suggests that it is not solely the amount of information exposed, but particularly the information content, that appears to be informative about the network’s behavior.

While gradient-based attribution usually led to higher attack AUCs, no significant difference was noticeable between gradient- and perturbation-based CLMs in metric-based and SVM-based attacks. However, the NN-based attacks clearly show that p-CLMs seem to be more informative for MIAs. This finding is supported by the fact that p-CLMs have been previously found to show a much stronger ability of concept localization as compared to g-CLMs (Lucieri et al., 2020b).

### 4.3 There is a need for differentially private concept-based explanations

The metric-based attack results indicated that DP successfully reduces the attack accuracy on traditional attack metrics such as the loss and class predictions. However, it highlights that the vulnerability added by exposing concept-based explanation metrics is mostly unaffected by DP. This finding is further reinforced by the results of the NN-based attacks, which clearly show that DP can even lead to higher model vulnerability when using concept-based explanations.

The most important reason for the ineffectiveness of DP when using concept-based explanations is the *post hoc* nature of the CAV method. Currently, there is no differentially private CAV procedure, which means that the binary concept classification layers are trained without any limitations on the differentially private network. This introduces new network parameters which can incorporate sensitive information about the data statistics of individual concept samples. Moreover, it is important to note that the CAVs have been trained only on the available concept subsets (*Derm7pt* for *ISIC* trained models, and *STARE* for *EyePACS* trained models). The fact that concept-based explanations were trained on extremely narrow datasets might have caused extreme variations in the explanation of the other data subsets, which lie outside the previously seen concept distribution. This even seems to be the case for CAVs computed on *EyePACS* trained models, which were computed on *STARE* while the MIAs were conducted on *ISIC* and *APTOS2019* subsets.

The conclusion of these results is two-fold. First, it is highlighted that there is a strong need for a differentially private variant of the concept-based CAV explanation method, which takes privacy constraints into account, even after the model is trained. Moreover, the results suggest that distribution shifts between concept training datasets and the remaining data distribution can potentially cause more harm, beyond unreliable concept predictions.

### 4.4 Concept-based explanations have no significant impact on model privacy in practice

The results in Section 3.3 clearly highlight that the practical vulnerability of the AI model to membership inference attacks is mostly independent of the choice of explanations exposed to stakeholders through the public API of a data-driven algorithm. Previous results from Shokri et al. (2021) already indicated that MIAs do not gain performance by utilizing attribution maps as the attack vector, but that this information can mainly be exploited by adversaries in tabular datasets with binary features. The findings in the present paper reinforce these previous results and demonstrate that image-based AI systems in biomedical domains are not significantly threatened by exposing attributions or concept-based explanations under the right deployment scenario. Furthermore, the membership inference attacks in this work were experimentally constructed as full white-box attacks for comparing the theoretical influence of different explanation techniques to the vulnerability of models. From a practical perspective, the presented results can therefore be interpreted as an upper bound on the potential vulnerability of explainable AI systems. Common deployment setups limit, for instance, the frequency of model requests allowed. This drastically complicates the acquisition of sufficient features for the training of attack models. Moreover, AI systems often provide processed values for prediction and explanation values instead of raw data. Although this reduces the sensitive information exposed by the victim model, previous works (Choquette-Choo et al., 2021) demonstrated that a post-processing of the prediction vector is not enough to fully eliminate privacy vulnerability.

To still assure a safe deployment of AI systems exposing concept-based explanations, a few points should be considered. First off, the conducted experiments assumed the full exposure of raw concept-based explanations. However, in a practical setting it might be more feasible to filter for only those concept explanations that are relevant to the current predictions. This could mean that not all CLMs are communicated as a matter of principle, but that CLMs are only presented for the concepts that were likely detected by the network. The same applies to the concept predictions scores. On one hand, this inconsistency of explanation output makes it harder for adversaries to collect relevant information for their attacks, and on the other hand reduces the explanation complexity for the relevant stakeholders consuming the explanations. Moreover, a post-processing of the raw concept predictions and CLMs is highly suggested to reduce the specific information leakage to overfitted model behavior. The main advantage of concept-based explanations as compared to feature attribution is that the explanation lies in the higher-level meaning, as compared to exact values or relations of quantities. Depending on the use case, it might make sense to blur, smoothen or add small amounts of noise to the CLM signal, as long as the actual interpretation by humans is not hampered. However, this is a trade-off that has to be carefully considered for each use-case separately, as it introduces strong assumptions about the network’s decision-making process.

In addition to safety measures on the side of explanations, it is even more important for model developers to ensure the integrity of the model’s training data, as well as the concept data used to train the CAVs. The experiments clearly show that a training dataset that is representative of a broad range of real-world data significantly decreases the probability of successful membership inference attacks. Although training datasets can never fully capture all variations of the real world, methods like data augmentation, style transfer, and the addition of various subsets of data acquired from a variety of sources and subpopulations are important tools towards robust training sets. Furthermore, various experiments highlighted the importance of clean, large, and representative concept datasets for CAV training. Concept datasets provide a communicative interface to the model’s representation of the target task and act as the human definition of the concepts at hand. Therefore, it is of crucial importance that these dataset samples are sufficiently representative of the concept’s variations, and that the dataset clearly and unambiguously describes the idea of the concept. The results indicate that current public datasets for concept training inherit serious biases and imbalances, which makes their utility debatable.

### 4.5 Differential privacy fails to defend against membership inference attacks

Most surprisingly, the experiment results indicated that training with DP did not only fail to defend against MIAs, but even reinforce the attack accuracies in some cases. As already discussed, the *post hoc* nature of concept-based explanations might introduce an exploitation to an already trained DP model. However, the results from Section 3.2 also illustrate that attack vectors composed of only target class predictions and attribution maps were more effective on models trained with differential privacy. Up to two-digit increases in attack performance were reported in some cases. The aim of DP is to provide a probabilistic guarantee on the privacy protection of individuals in a dataset. One possible reason for the reported behavior is, that the dataset distributions in the suboptimal deployment scenario were so different that the MIAs on DP trained models did not reveal the presence of actual individuals, but their subgroups. DP training, in theory, forces the network to generalize upon the broad data distribution of the training set, without focussing too much on single examples. This also means that some underrepresented training samples from marginal distributions, are considered less as in the unrestricted training, to avoid over fitting on these examples. It might be possible that the DP training allows models to focus more on a more general core distribution, while neglecting marginal distributions. This might potentially lead to more sensitive responses of its internal representation when exposed to unseen data distributions, and requires further investigation.

### 4.6 CAVs alignment and performance suffer from private training

The results presented in Section 3.4 indicate that there is a notable impact of DP on the generated CAVs. For most of the datasets, DP led to a decrease in average CAV accuracy and an increase in standard deviation. For the synthetic *SCDB* dataset, this is further confirmed by the cosine similarities of repeated CAV computation with different random undersampling of the concept training dataset.

Both biomedical image datasets, however, showed a small increase in similarity of CAVs when trained in the DP setting. Interestingly, the average CAV accuracies and the CAV alignment do not correlate with the models’ test performances in those datasets. *ISIC*, for instance, suffered from a significant decline in performance for all architectures when trained in the DP setting, but the average CAV accuracy was not impacted to a significant extent. For *SCDB*, on the other hand, a strict correlation between concept quality and model performance can be observed. A possible reason for this unintuitive behavior is the imperfection of the definition of concepts in real-world biomedical domains, in conjunction with potential biases due to non-representative concept training sets. The fact that CAV accuracies remain unchanged when the target task performance decreases, either indicates that the concepts were prioritized in favor of other, more irrelevant features, or that the concepts as defined by the concept dataset mainly encode spurious correlations instead of the actual concept at hand.

### 4.7 The vulnerability to privacy attacks is architecture dependent

The experimental results overall indicate that victim models based on the *ConvNeXt* architecture are slightly more vulnerable compared to *ResNet50* and *NFNet*. This trend is reinforced by observing the mean attack accuracies on the attack vector composed of target class predictions, concept predictions and g-CLMs (*TP + CP + g-CLMs*) in NN-based attacks for both datasets, where *ConvNeXt* scores an average of 86.56%, followed by *NFNet* with 84.58% and *ResNet50* with 79.11%. Although *ResNet50* seems less vulnerable in this perspective, the difference to *NFNet* vanishes when considering different attack vector combinations. The trend of *ConvNeXt* being most vulnerable, however, is consistent over all metric-based, SVM-based and NN-based settings. This indicates, that the vulnerability of a model to MIA attacks is indeed not only dependent on the training dataset, but also on the architecture at hand.

### 4.8 Limitations

Despite the careful design of experimentation, some limitations of this work remain. Due to the high complexity of optimization problems with high-dimensional feature vectors, the convergence of NN-based MIAs cannot be guaranteed. For this large-scale benchmark, a fixed optimization scheme with early stopping was chosen to get reasonably consistent and comparable results. However, some attacks might be further improved when specifically focusing on their optimization. For the SVM-based attacks, dimensionality reduction has been conducted for high-dimensional attack vectors, including attributions and CLMs. This reduction might have also had a slight influence on the results achieved by these attacks. Although a theoretical comparison of models trained with different optimization schemes (e.g., baseline vs. DP) is possible, the practical relevance remains uncertain. Especially in the case of DP trained medical image models, it is unavoidable that models achieve lower performances, due to comparatively high class imbalances. Moreover, DL models can have substantial differences in their decision-making, even when model performances appear to be comparable. This work investigates the vulnerability of explainable AI systems in suboptimal and optimal optimization settings. However, the data distributions and data shifts can vary greatly in different real-world deployment scenarios. Therefore, it is advised to evaluate each particular deployment case on its own, using this work as a guideline. The theoretical analysis of white-box MIAs served as an empirical upper-bound to the vulnerability of explainable AI models. However, most DL models deployed in biomedical contexts will allow only query-limited black-box access through a prediction and explanation API. The real vulnerability is therefore expected to be lower, as the training of shadow models would be required to perform the final MIA. Whereas Saliency is a non-parametric attribution method, the quality of Occlusion maps can vary considerably with the choice of parameters. The results for attribution-based MIAs can therefore vary depending on the selected parameters. In this work, parameters for Occlusion have been chosen upon experience, and optimizing the subjective quality of attribution maps. An even stronger limitation is imposed by CAV explanations, as the quality of concept-based explanations not only depends on the parameter choice, but also on the available concept training data. Larger and higher quality concept datasets could potentially lead to higher quality explanations that might result in increased privacy vulnerability.

## 5 Conclusion

In a row of systematic experiments, the theoretical effect of concept-based explanations on the vulnerability of models under membership inference attack is investigated. Using a synthetic concept dataset in addition to two realistic biomedical examples of skin lesion analysis and diabetic retinopathy detection, the vulnerability of three different model architectures exposing varying levels of explanations is assessed. The results suggest that concept predictions and CLMs expose more sensitive information as compared to traditional input feature attributions, potentially leading to higher model vulnerability against membership inference attacks in suboptimal deployment scenarios. However, further analysis highlights the insignificance of this threat for realistic application scenarios of biomedical imaging and emphasizes the importance of representative and balanced training datasets, as well as extensive data augmentation. Moreover, a need for a differentially private training procedure for concept-based explanations is identified. In addition, the results revealed that training with differential privacy does not necessarily lead to an improved resilience against membership inference attacks, but that it sometimes even reinforces privacy leaks in imbalanced datasets as commonly used in the biomedical domain. The computation of concept-based explanations, on the other hand, is negatively influenced by the constraints introduced by differential privacy.

This work opens up a series of further interesting questions. In this work, we specifically focussed on investigating the impact of concept-based explanations on white-box membership inference attacks. To fully investigate the privacy risk of explanations, however, it is necessary to also evaluate the vulnerability against other types of attacks, including model extraction and input reconstruction attacks. Moreover, even though we showed that concept-based explanations, in practice, do not introduce significant vulnerabilities for MIAs, other scenarios must be further investigated. Assuming that concept-based explanations potentially improve model extraction attacks, more complex attack scenarios still need to be investigated. For example, it would be interesting to explore how model extraction attacks could be exploited by adversaries to improve their MIAs by subsequent metric-based attacks on the loss. Besides local explanations in the form of concept predictions and CLMs, the CAV method can provide further statistics such as the TCAV score. Future work should investigate the privacy risk posed by the application of other human-centric explanation methods, including the utilization of the TCAV score, as well as their combination, to properly reflect practical application scenarios. Yet, the impact of privacy-preserving methods on the quality of explanations has only been numerically measured by approximating subjective values such as smoothness and continuity. However, the process of explaining a model decision is highly dependent on the interpretation of facts by the explainee. Thus, future works should focus on user studies to properly quantify the subjective decrease in explanation quality in real application scenarios. Lastly, this work identified that the TCAV method itself is, so far, incompatible with the framework of differential privacy. Moreover, the usually low number of concept samples makes it particularly challenging to align concept training with notions of privacy. Future work should investigate this problem, deriving ways to allow privacy-aligned concept training in low data regimes.

Safety and accountability are two major aspects in the deployment of modern AI systems in high stakes domains such as healthcare and biomedicine. Transparency and privacy follow two opposing goals in decision-making. Therefore, it is of utmost importance to thoroughly investigate their interplay to ensure the optimal trade-off for the given application at hand.

## Data Availability

Publicly available datasets were analyzed in this study. This data can be found here: ISIC: https://challenge.isic-archive.com/data/. Derm7pt: https://derm.cs.sfu.ca. EyePACS: https://www.kaggle.com/datasets/mariaherrerot/eyepacspreprocess. STARE: https://cecas.clemson.edu/∼ahoover/stare/. APTOS2019: https://www.kaggle.com/datasets/mariaherrerot/aptos2019. SCDB: https://zenodo.org/record/6258557.

## Footnotes

1 The ISIC Archive is accessible at https://www.isic-archive.com

2 The *Derm7pt* dataset is accessible at https://derm.cs.sfu.ca/

3 The *EyePACS* dataset is accessible at https://www.kaggle.com/datasets/mariaherrerot/eyepacspreprocess

4 The *STARE* dataset is accessible at https://cecas.clemson.edu/∼ahoover/stare/

5 The *APTOS2019* dataset is accessible at https://www.kaggle.com/datasets/mariaherrerot/aptos2019

6 The *SCDB* dataset is accessible at https://zenodo.org/record/6258557

7
https://timm.fast.ai/

8
https://opacus.ai/

9
https://captum.ai/
